# Evaluation of the efficacy of hearing aids in older adults: a multiparametric longitudinal study protocol

**DOI:** 10.1186/s12877-021-02033-z

**Published:** 2021-02-05

**Authors:** Domenico Cuda, Sara Ghiselli, Alessandra Murri

**Affiliations:** ENT Department, Ospedale Guglielmo da Saliceto, Via Cantone del Cristo 40, 29121 Piacenza, Italy

**Keywords:** Hearing aids, Hearing‐related healthy aging, Hearing loss in the older adults, Presbyacusis

## Abstract

**Background:**

Prevalence of hearing loss increases with age. Its estimated prevalence is 40–50 % in people over 75 years of age. Recent studies agree that declinein hearing threshold contribute to deterioration in sociality, sensitivity, cognition, and quality of life for elderly subjects.

The aim of the study presented in this paper is to verify whether or not rehabilitation using first time applied Hearing Aids (HA) in a cohort of old people with hearing impairment improves both speech perception in a noisy environment over time and the overall health-related quality of life.

**Methods:**

The monocentric, prospective, repeated measurements, single-subject, clinical observational study is to recruit 100 older adults, first-time HA recipients (≥ 65 years).The evaluation protocol is designed to analyze changes in specific measurement tools a year after the first HA usage in comparison with the evaluation before HA fitting.

Evaluations will consist of multiparametric details collected through self-report questionnaires completed by the recipients and a series of commonly used audiometric measures and geriatric assessment tools. The primary indicator of changes in speech perception in noise to be used is the Italian version of Oldenburg Satz (OLSA) test whereas the indicator of changes in overall quality of life will be the Assessment of Quality of Life (AQoL) and Hearing Handicap Inventory for the Elderly (HHIE) questionnaires. The Montreal Cognitive Assessment (MoCA) will help in screening the cognitive state of the subjects.

**Discussion:**

The protocol is designed to make use of measurement tools that have already been applied to the hearing-impaired population in order to compare the effects of HA rehabilitation in the older adults immediately before first HA usage (Pre) and after 1 year of experience (Post). This broad approach will lead to a greater understanding of how useful hearing influences the quality of life in older individuals, and therefore improves potentials for healthy aging. The data is to be analyzed by using an intrasubject endpoint comparison. Outcomes will be described and analyzed in detail.

**Trial registration:**

This research was retrospectively registered underno. NCT04333043at ClinicalTrials.gov (http://www.clinicaltrials.gov/) on the 26 March 2020. This research has been registered with the Ethics Committee of the Area Vasta Emilia Nord under number 104, date of approval 17/07/2017.

## Background

Hearing loss is one of the most common disabilities in the world. Prevalence of this disease increases with age and its estimated prevalence is 40–50 % in people older than 75 years [1].

The term presbyacusis refers to decreased hearing sensitivity in older adults subjects. Nevertheless, this term has a broader meaning including all types of hearing loss that occur in the older adults. Presbyacusis also involves genetic age-related cumulative effects and potential secondary injuries to the auditory system (noise-induced hearing loss, ototoxicity, etc.).

Presbyacusis is the main cause of stable hearing loss and the most common disablingdisease in the older adults. [2].

Presbycusis is characterized by a bilateral symmetrical neurosensorial hearing loss that initially involves high frequencies and secondarily medium and low frequencies. Auditory threshold modifications are related to variations in detection, localization and speech discrimination especially in noisy environments [3, 4]. These variations deteriorate sociality, sensitivity, cognition, and quality of life of the subject [5–7].

However, cognitive abilities can benefit from the hearing-impaired subject using a hearing aid (HA). It is clear that hearing loss and cognitive abilities are linked and influence each other [8, 9].

Periodic monitoring of the hearing threshold is essential in early identification of communication disability or handicap, and the initiation of early treatment.

Since presbycusis is a multifactorial disease, the decline of the auditory threshold can be reduced by limited noise exposure, medical treatment in case of systemic or ear diseases or, in most cases, by using a HA [10–12].

Various studies have shown that HA improve quality of life (QoL) and cognitive functions in patients with presbycusis [8, 13–15]. Compared to patients with untreated hearing loss, patients with HA show improvements in social and emotional scores, communication and cognition skills, and do not suffer so much from depression [16].

In most studies, HA benefit is investigated using traditional clinical audiometric tests or self-assessed outcomes [17]. Only a few studies use multiparametric experimental protocols that explore both audiometric and cognitive skills improvement in older hearing-impaired subjects with HA [7].

In a recent study, Tognola et al. investigated HA benefit in the older adults using complete multi-parametric protocol. This study shows a significant correlation between auditory outcomes, hearing impairment, and cognition but it examined subjects once (a year after the first fitting of HA) and it did not investigate the role played by HA use in maintaining long-term health outcomes. [18].

The present paper describes the first application of the protocol design of a monocentric prospective longitudinal study in a large cohort of older adults with HA. Its unique features include severaltests that include evaluation of hearing aid benefit in a noisy environment and simultaneous assessments of the long-term evaluation of the mental, psychosocial, and QoL domains.

## Methods and design

### Study aims

The primary aim of this study is to identify whether or not there is any significant improvement in speech recognition in noise in older adults patients ( > = 65 years) who are using HA for the first time. This improvement is to be verified by using a Signal to Noise Ratio (SNR) reduction which will be evaluated using the OLSA test.The secondary aim is to process the correlations between the use of the HA and quality of life and cognitive status in older adults. Any improvement or deterioration can be seen in higher or lower scores in auto-assessment questionnaires.

### Study design

This is a prospective observational, repeated measurement, single-subject design study.

Changes in speech recognition by noise score, health-related quality of life, and overall well-being are assesses.

The study design is monocentric and is conducted at the outpatient service of the ENT Department of the “Saliceto” Hospital in Piacenza, Italy.

All the materials used are appropriate for the italian language.

This study is registered with the Ethics Committee of the Area Vasta Emilia Nord under number 104; date of approval 17/07/2017.

### Timing schedule

The study protocol assessments coincides with the routine clinical examinations. Full test-battery and questionnaires are administered in both examinations: one before the HA fitting (Pre) and the other after one year of HA use (Post).

A short description of the procedure is shown in Table 1 and listed below.

**Table 1:** procedure and assessment tools used at different time of evaluation

	Enrolment	Post-allocation	
**TIMEPOINT**	***t***_***0***_	***Pre***	***Post***
**ENROLMENT**			
** Eligibility screen**	X		
**Informed consent**	X		
**Allocation**	X		
**AUDIOMETRIC ASSESSMENTS**			
***unaided pure tone audiometry***		X	X
***unaided speech audiometry***		X	
***aided speech audiometry***			X
***test of the HA functioning***			X
***OLSA test***		X	X
***SELF-ASSESSMENTS QUESTIONNAIRES***			
***MOCA***		X	X
***HHIE-S***		X	X
***AQoL***		X	X
***IOI-HA***			X

Baseline (t0):

- Signature of the Patient giving Informed Consent..

First assessment (Pre):

- otoscopy and cleaning of the external auditory canal (if required).
- unaided pure tone audiometry (right and left ear).
- unaided speech audiometry in quiet (right and left ear).
- Italian version of Oldenburg Satztest (OLSA test) [19].
- Self-assessment questionnaires:
- Montreal Cognitive Assessment (MoCA) [20].
- Hearing Handicap Inventory in the Elderly Screening test (HHIE-S) [21].
- Assessment of Quality of Life *(*AQoL) [22].
- Abbreviated Profile of Hearing *A*id Benefit (APHAB) [23]..

Second assessment (Post):

- otoscopy and cleaning of the external auditory canal (if required).
- test of the HA functioning.
- unaided pure tone audiometry (right and left ear).
- aided speech audiometry in quiet (in free field).
- Italian version of OLSA test.
- Self-assessment questionnaires:

- MoCA.
- HHIE-S
- AQoL.
- International Outcome Inventory for Hearing Aids (IOI-HA) [24].
- APHAB in aided condition.

To complete all the measures takes approximately one hour: 30 minutes for performing the standard self-report questionnaires and 30 minutes for the other tests.

### Subjects

Study subjects who use unilateral or bilateral hearing aids for the first time are to be included. Eligible participants are consecutively identified on the HA registry of the Local Unit of the National Health System (NHS). The Italian NHS is a welfare system which totally or partially funds the HA costs of the patients selected.

Subjects are > = 65 years old using unilateral or bilateral HA for the first time. According to local NHS threshold-criteria for funding adult HAs, the best ear of the patients must have a Pure-Tone Average (PTA) > = 45 dB HL. PTA is the average air-tonal threshold at 500, 1000, 2000, and 4000 Hz frequencies.

Only the subjects who signed the Patient Informed Consent Form prior to the first assessment (baseline, t0) are eligible for the clinical investigation.Approximately 100 individuals will be included. The sample size of 100 people was recruited according to a previous preliminary study by the authors (Tognola et al. 2019) and depending on olderpopulation eligible to utilize HA. Indeed, eligible participants are consecutively identified in the HA registry of the Local Unit of the National Health System (NHS).

#### Selection Criteria

Inclusion:

- Age > = 65 years.
- First use of unilateral or bilateral HA.
- HA partially or totally funded by the Italian National Health System.
- Willingness to participate in.
- Willingness to comply with all study procedures.
- Fluency in italian language used to assess clinical performance.
- Able to decide on study participation personally.
- Able to independently sign their consent..

Exclusion:

- Unilateral hearing loss.
- Previous use of HA.
- Significantly/severely dependent or fragile.
- Unable to provide consent personally.
- Unable to complete questionnaires for self-assessment independently.
- Significant comorbidities preventing study participation.
- Subjects with unrealistic expectations on benefits, risks and limitations concerning the procedure and prosthetic device..

### Materials

Changes in the hearing aid benefit and the overall health status of the older adults at post-HA use are evaluated by a selection of observational clinical assessment tools frequently used in audiology and/or geriatric practices.

All questionnaires have been validated for the Italian language.

Expert personnel have been designated to manage the administration of the questionnaires in order to understand whether or not the subject clearly understands what the task involves.

A short description of the administered assessments is listed alphabetically below (see Table 2).

**Table 2:** Healthy-ageing domains and assessment tools used for evaluation

Domain	Clinician report	Self-report by recipient			Routine Audiology		
	MOCA	AQoL	IOI-HA	HHIE-S	PTA	SRT	OLSA Test
Hearing					◆	◆	◆
Emotional			◆	◆			
Cognition	◆						
Loneliness / Social Isolation			◆	◆			
General Health		◆	◆				
Quality of Life		◆	◆				

#### Abbreviated Profile of Hearing Aid Benefit (APHAB)

Abbreviated profile of Hearing Aid Benefit (APHAB) is the abbreviated version of the Profile of Hearing Aid Benefit (66 items).

APHAB is a self-assessment questionnaire composed of 24 questions that assess the advantage and the communicative problems of HA use [25].

The data obtained provides information about several typical workday situations considering four subscales: Ease of Communication, Reverberation, Background Noise, and Aversiveness of Sounds.

A low APHAB score indicates better performances. A measurement of the benefit will be calculated by subtracting APHAB scores in Post evaluation (with HA) from Pre evaluation.

The Italian translation of the APHAB questionnaire is available on the Hearing Aid Research Lab (HARL) web site at (https://harlmemphis.org/wp content/uploads/2020/05/ITALIAN.pdf) [23].

#### Assessment of Quality of Life (AQoL-8D)

The Assessment of Quality of Life (AQoL) was designed for use across health conditions to enable evaluation studies of health economics. AQoL assesses the impact of interventions on health-related quality of life by comparing different disease settings and monitoring longitudinal changes in a broad range of health conditions [26–28].

The AQoL-8D consists of five psychosocial and three physical dimensions. The three physical dimensions are related to a single construct (the ‘physical super-dimension’) and the five psychosocial dimensions are related to a single construct (the ‘mental super-dimension’).

The test includes 35 items with 5 possible answers.

The Italian translation of the AQoL questionnaire is available on the AQoL web site (www.aqol.com.au) [22].

#### Audiometric Assessments (Routine)

These include standard threshold measurements for frequencies of 250–8000 Hz (pure tone audiogram) and speech discrimination (speech audiometry) in a quiet situation. The pure tone audiometryis performed in a soundproof room using a Madsen Astera (by Natus Medical Incorporated, Denmark) audiometer and TDH39 supra-aural earphones.

The pure tone audiometryunder earphone is assessed in Pre and Post evaluations, and in unaided (dB HL) condition. Speech audiometry is assessed in Pre-evaluation under earphone whereas it is assessed in the free field in aided (dB SPL) condition in Post evaluation.

Speech audiometry shows the Speech Reception Threshold (SRT), defined as the level of speech corresponding to 50 % of correct answers. In the SRT test, the speech stimuli (list of disyllabic words) [29] are pre-recorded and delivered by a loudspeaker at zero degrees azimuth in the sound field.

The subject is asked to repeat a list of 20 words (phonetically balanced) and the percentage of correctly repeated words is evaluated for each intensity-level presented. The intensity ranges between 30 and 80 dB HL and every word list is presented at an intensity of 10 dB higher than the previous one. An intelligibility curve is obtained and SRT is derived by curve interpolation.

#### Hearing Handicap Inventory in the Elderly Screening test (HHIE-S)

The Hearing Handicap Inventory for the Elderly Screening Version (HHIE-S) is a self-assessment scale developed to assess the effects of hearing impairment on emotional and social adjustment in everyday life of the older adults [30–33].

This test contains a 10-item questionnaire. A low HHIE-S score indicates fewer perceived problems.

The Italian version of this material is under publication [21].

#### International Outcome Inventory for Hearing Aids (IOI-HA)

The International Outcome Inventory for Hearing Aids (IOI-HA) is a self-report questionnaire developed to quantify the satisfaction of hearing aid users and the impact these devices have on their lives. The IOI-HA contains seven domains:(1) the time for whichHAhave been used;(2) benefit; (3) residual limitation in daily life activities; (4) satisfaction; (5) residual restrictions to participation; (6) impact on other people; (7) quality of life. The answers to each question range from poor performance (1) to best performance (5) [34, 35].

A high score is correlated with a good outcome in aided conditions.

Cox et al. were responsible for the Italian validation of this questionnaire [24].

#### Montreal Cognitive Assessment (MoCA)

Montreal Cognitive Assessment (MoCA) is a cognitive screening instrument used to detect mild cognitive impairment, a high-risk condition for dementia forms [20, 36–38].

It is composed of 12 subtasks assessing different cognitive skills, including short-term and delayed verbal memory, executive function, and attention. The total MoCA score ranges from 0 (worst performance) to 30 (best performance). Mild cognitive dysfunction is suspected when the final score is less than 26.

Different strategies will be integrate by clinicians in order to avoid a misdiagnosis or overdiagnosis of cognitive impairment. Personnel will be trainedto administrationof this tool in patients with hearing loss. In particular, they will assess the correct use of hearing aids; they will ensure that the test environments meet standards for ambient noise levels and they can use alternative presentation modality of the items with auditorycontents (with multimodal presentation: both auditory and visual presentation) [39].Santangelo et al. produced the normative data for the Italian population [20].

#### OLSA test

The OLSA test (HörTechGmbH, Germany) is a versatile examination that is structured into two randomized lists of 30 sentences of five-word, semantically unpredictable [40]. This tool is administered after a training session to minimize the learning curve. The test is carried out using a closed-set response format.

The test yields the Signal to Noise Ratio (SNR) measurement in dB at which the subject recognizes 50 % of the words presented.

The test is administered using the S0N0 presentation setup (speech and noise from the same frontal loudspeaker).The background noise is presented at 65 dB SPL whereas the speech level is adaptively adjusted depending on the subjects’ response to obtain the SNR.

Puglisi et al. (2015) described the reference ranges and standard deviations of the OLSA test for the Italian language by determining different levels to identify at-risk patients [19].The cutoff of the SNR dB (SRT) among the older adults was set to – 0.4 dB based on a reference mean level of -6.7 plus 2 standard deviations [19].

### Statistical considerations:

An intrasubject endpoint comparison is to be used for primary and secondary study objectives. The pairwise comparisons are of interest: previous use of HA (Pre)-to-12 months HA use (Post).

Investigation will apply multivariate exploratory factor analysis to reduce the number of variables and identify possible latent factors, followed by Spearman correlation to analyze the relationships between the variables and the factors.Finally regression analysis will apply to investigate how factors and variables relate to each other and how they predict outcomes.

Data will be analyzed by nonparametric tests for categorical variables and by t-test for continuous variables.

Questionnaires analysis will be performed by using a nonparametric test.

## Discussion

This study aims to show the potential positive effect of HAs on auditory skills and on quality of life of older adults with hearing loss. In particular, the efficacy of HA in speech perception in noisy environments and the improvement of quality of life in the older adults using HA for the first time is to be evaluated. The broad approach will lead to a greater understanding of how hearing impacts the quality of life in older adults thereby improving healthy aging.

Since the older adults are increasing in number in the Italian population, a greater number of hearing aids will be required to treat this growing presbycusic population. This will strongly affect the finances of the National Health System, which bears the full or partial cost of HA in most cases. This is also a good reason to understand the real benefits of the HA application clearly. The study outcomes are intended to provide transparent and comparable evidence-based information for healthcare policymakers by supporting the provision of health services for the treatment of hearing loss.

The comprehensive protocol considers a range of widely accepted, interrelated metrics associated with aging in addition to functional hearing. The use of this protocol does not propose the investigation of causal effects but rather investigation of the pre-post HA candidates by observing long-term health outcomes.

The primary endpoint of this study is to identify significant improvement in speech recognition in noise. The percentage of subjects with SNR > 1.5 dB at OLSA test is to be evaluate in order to verify the endpoint.

The OLSA test is the tool most frequently used to find the auditory outcome in noisy environment in the majority of the recent literature. Use of this test has overcome the traditional speech audiometry because it uses sentences consisting of a matrix of 5 semantically unpredictable words and is based on an adaptive method. This has overcome the memory capacity of HA users who perform speech audiometry several times at follow up and are able to learn the words in the lists. Furthermore, in contrast to speech audiometry with sentences of common use that have a semantic context, the OLSA test does not have a predictable semantic context.

During this study two-time point, tonal unaided audiometry was used to check any auditory threshold modification and worsening of the audiometric clinical situation over time. Speech audiometry in unaided conditions in the first assessment and in aided conditions in the second assessment were used because it describes the sample and allows it to be compared to the existing literature.

The second objective of this study is based on the evaluation of possible improvements in the overall health-related quality of life in the sample.

To verify this aim, self-report questionnaires were chosen, which are essentially non-verbal and consequently remove challenges directly associated with utilizing new HA. The percentage of subjects who improve the questionnaire score using HA (second assessment) compared to the first evaluation in unaided conditions is to be evaluated.

Improvement in quality of life can be evaluated by using different questionnaires. The AQoL questionnaire is used in this study because of its robust psychometric characteristics and wide diffusion in the health-related quality of life literature. In a review by Mihalopoulos et al., (thatanalyzesinstruments for depression outcome measurements) report that AQoL-8D “had the highest correlation with the disease-specific measurements and the best goodness-of-fit transformation properties” [28].

The HHIE has been included in the study in order to better understand the effects of hearing impairment on emotional and social adjustment in everyday life. It is one of the most widely applied questionnaires with respect to auditory participation and has been reported with both HA and cochlear implant recipients [41]. A significant reduction of the questionnaire score in Post vs. Pre-evaluation is expected in this study, which suggests quality of life improvement.

The MoCA questionnaire was chosen to screen the cognitive abilities of subjects. The use of this questionnaire will exclude deficits in these abilities. Using this test in Pre and Post evaluation will verify that cognitive abilities do not affect other questionnaires and HA results. The use of the MoCA questionnaire in the Pre and Post evaluation will exclude a reduction of the cognitive abilities over time. A reduction of these abilities can be a sign of the deterioration of the general state of health that may affect the execution of the other questionnaires used. The same score is expected in Pre and Post evaluation of the same subject.

The APHAB questionnaire is used to assess problems in speech understanding in different listening situations. This self-assessment is one of the most used questionnaires in clinical practice. An improvement in the scores in Post evaluation compared to Pre evaluation will verify a reduction of listening problems secondary to HA usage.

The IOI-HA questionnaire is only used in the Post evaluation. It will help understand the difficulties and benefits of HA use perceived by the patients. In particular, this questionnaire will give us a picture of the HA outcomes in different areas. Furthermore, being a standardized questionnaire, it will facilitate comparison of the sample with the existing literature.

The strength of this study lies in evaluating the auditory and health outcomes over time which contrasts with most of the recent studies that show an overview of the outcomes at a precise moment and various other biased sources.

Furthermore, as reported by Hanratty and Lawlor, people over 70 are thought to have a hearing impairment that would benefit from a HA but a high percentage of these people probably never use their aid [12]. The study presented in this paper can not only underline the benefits but also real HA usage over time. This information is very important for healthcare policy because the Italian National Health System bears all or some of the HA costs for most of these patients.

These outcomes should contribute to providing key actors of the health system with means of enhancing their part in a collective endeavor targeting the best care and quality of life for older adults citizens because general well-being translates into healthy aging.

The single-subject, repeated-measures design allows for subjects to serve as their own controls, and thus increases statistical power. The relatively large population size helps provide a good estimate of effect size and would make the results broadly applicable.

## Data Availability

Data can be obtained by the corresponding author upon request.

## Abbreviations

APHAB: Abbreviated Profile of Hearing Aid Benefit
AQoL: Assessment of Quality of Life
IOI-HA: International Outcome Inventory for Hearing Aids
HA: Hearing Aids
HHIE-S: Hearing Handicap Inventory in the Elderly Screening test
MoCA: Montreal Cognitive Assessment
PTA: Pure Tone Audiometry
SRT: Speech Reception Threshold

## References

[1] World Health Organization. Global estimates on prevalenceof hearing loss. Geneva, Switzerland: World Health Organization2012. http://www.who.int/pbd/deafness/WHO_GE_HL.pdf?ua=1.

[2] Allen PD, Eddins DA (2010). Presbycusis phenotypes form a heterogeneous continuum when ordered by degree and configuration of hearing loss. Hear Res.

[3] Divenyia P, Stark PB, Haupt KM (2005). Decline of speech understanding and auditory thresholds in the elderly. J Acoust Soc Am.

[4] National Institute for Health and Clinical Excellence (NICE). 2018. NICE guideline 2018: Hearing loss in adults: assessment and management. Published: 21 June 2018 (accessed 08 October 2020). Available from: www.nice.org.uk/guidance/ng98 30011159

[5] Fortunato Forli S, Guglielmi F, De Corso V, Paludetti E, Berrettini G, Fetoni S. AR. A review of new insights on the association between hearing loss and cognitive decline in ageing. Acta Otorhinolaryngol Ital. 2016;36:155 – 66. 10.14639/0392-100X-993. 10.14639/0392-100X-993 PMC4977003 27214827

[6] Uhlmann RF, Larson EB, Rees TS, Koepsell TD, Duckert LG (1989). Relationship of hearing impairment to dementia and cognitive dysfunction in older adults. JAMA.

[7] Moore D, Edmondson-Jones M, Dawes P, Fortnum H, McCormack A, Pierzycki RH, Munro KJ (2014). Relation between speech-in-noise threshold, hearing loss and cognition from 40–69 years of age. PLoS One.

[8] Meister H, Rählmann S, Walger M, Margolf-Hackl S, Kießling J (2015). Hearing aid fitting in older persons with hearing impairment: the influence of cognitive function, age, and hearing loss on hearing aid benefit. Clin Interv Aging.

[9] Rönnberg J, Lunner T, Ng EH, Lidestam B, Zekveld AA, Sörqvist P, Lyxell B (2016). Hearing impairment, cognition and speech understanding: exploratory factor analyses of a comprehensive test battery for a group of hearing aid users, the n200 study. Int J Audiol.

[10] De Benedetto M, Cuda D. La terapia protesica nell’anziano. In Grande F, Leone CA, editors.La patologia dell’orecchio nell’anziano.Pisa, Pacini Editore; 1996. p. 183–201.

[11] Mulrow CD, Tuley MR, Aguilar C (1992). Sustained benefits of hearing aids. J Speech Hear Res.

[12] Hanratty B, Lawlor DA (2000). Effective management of the elderly hearing impaired: a review. J Public Health Med.

[13] Lunner T (2003). Cognitive function in relation to hearing aid use. Int J Audiol.

[14] Lunner T, Sundewall-Thorén E (2007). Interactions between cognition, compression, and listening conditions: effects on speech-in-noise performance in a two-channel hearing aid. J Am Acad Audiol.

[15] Brodie A, Smith B, Ray J (2018). The impact of rehabilitation on quality of life after hearing loss: a systematic review. Eur Arch Otorhinolaryngol.

[16] Lopez-Poveda EA, Johannesen PT, Pérez-González P, Blanco JL, Kalluri S, Edwards B (2017). Predictors of hearing-aid outcomes. Trends Hear.

[17] Wattamwar K, Qian ZJ, Otter J, Leskowitz MJ, Caruana FF, Siedlecki B (2017). Increases in the rate of age-related hearing loss in the older old. JAMA Otolaryngol Head Neck Surg.

[18] Tognola G, Mainardi A, Vincenti V, Cuda D (2019). Benefit of hearing aid use in the elderly: the impact of age, cognition and hearing impairment. Acta Otorhinolaryngol Ital.

[19] Puglisi GE, Warzybok A, Hochmuth S, Visentin C, Astolfi A, Prodi N (2015). An Italian matrix sentence test for the evaluation of speech intelligibility in noise. Int J Audiol.

[20] Santangelo G, Siciliano M, Pedone R, Vitale C, Falco F, Bisogno R (2015). Normative data for the montreal cognitive assessment in an Italian population sample. Neurol Sci.

[21] Cocchi C.Validazione in lingua italiana dell’ Hearing Handicap Inventory for the Elderly-Screening Version (HHIE-S) e sue correlazioni con le disabilità nella comunicazione in età geriatrica.https://morethesis.unimore.it/theses/available/etd-09182017-122810/Accessed 04 Mar 2020.

[22] Centre for Health Economics. University M. AQoL-8D Data Collection Copy. http://www.aqol.com.au/documents/translations/AQoL-8D_copia_raccolta_dati.pdf. Accessed 05 Mar 2020

[23] Hearing Aid Research Lab (HARL).APHAB Questionnaires. https://harlmemphis.org/wp-content/uploads/2020/05/ITALIAN.pdf. Accessed 07 Oct 2020.

[24] Cox RM, Stephens D, Kramer SE (2002). Translations of the International Outcome Inventory for Hearing Aids (IOI-HA). Int JAudiol.

[25] Cox RM, Alexander GC (1995). The abbreviated profile of hearing aid benefit. Ear Hear.

[26] Hawthorne G, Richardson JR, Day NA (2001). A comparison of the Assessment of Quality of Life (AQoL) with four other generic utility instruments. AnnMed..

[27] Maxwell A, Özmen M, Iezzi A, Richardson JR (2016). Deriving population norms for the AQoL-6D and AQoL-8D multi attribute utility instruments from web-based data. Qual Life Res.

[28] Mihalopoulos C, Chen G, Iezzi A, Khan MA, Richardson JR (2014). Assessing outcomes for cost-utility analysis in depression: comparison of five multi-attribute utility instruments with two depression-specific outcome measures. BrJ Psychiatry.

[29] Turrini M, Cutugno G, Maturi P, Prosser S, Leoni FA, Arslan E (1993). Bisyllabic words for speech audiometry; a new Italian material. Acta Otorhinolaryngol Ital.

[30] Newman C, Jacbson G, Hug G, Weinstein B, Malinoff R (1991). Pratical method for quantifying hearing aid benefit in older adults. J Am Acad Audiol.

[31] Ventry I, Weinstein B (1983). Identification of elderly people with hearing problems. ASHA.

[32] Lichtenstein MJ, Bess FH, Logan SA (1988). Validation of screening tools for identifying hearing-impaired elderly in primary care. JAMA.

[33] Weinstein BE, Ventry IM (1983). Audiometric correlates of the Hearing Handicap Inventory for the elderly. J Speech Hear Disord.

[34] Cox RM, Alexander GC (2002). The International Outcome Inventory for Hearing Aids (IOI-HA): psychometric properties of the English version. Int J Audiol.

[35] Cox R, Alexander G, Beyer C (2003). Norms for the international outcome inventory for hearing aids. J Am Acad Audiol.

[36] Nasreddine Z, Phillips N, Bédirian V, Charbonneau S, Whitehead V, Collin I (2005). The Montreal Cognitive Assessment, MoCA: a brief screening tool for mild cognitive impairment. J Am Geriatr Soc.

[37] Conti S, Bonazzi S, Laiacona M, Masina M, Coralli MV (2015). Montreal cognitive assessment (MoCA)-Italian version: regression based norms and equivalent scores. Neurol Sci.

[38] Siciliano M, Chiorri C, Passaniti C, Sant’elia V, Trojano L, Santangelo G (2019). Comparison of alternate and original forms of the Montreal Cognitive Assessment (MoCA): an Italian normative study. Neurol Sci.

[39] Dupuis K, Pichora-Fuller MK, Chasteen AL, Marchuk V, Singh G, Smith SL (2015). Effects of hearing and vision impairments on the Montreal Cognitive Assessment. Neuropsychol Dev Cogn B Aging Neuropsychol Cogn.

[40] Houben R, Koopman J, Luts H, Wagener KC, VanWieringen A, Verschuure H (2014). Development of a Dutch matrix sentence test to assess speech intelligibility in noise. Int J Audiol.

[41] Manrique-Huarte R, Calavia D, Huarte Irujo A, Girón L, Manrique-Rodríguez M (2016). Treatment for Hearing Loss among the Elderly: Auditory Outcomes and Impact on Quality of Life. Audiol Neurootol.

